# Dr. Good's Study of Medicine

**Published:** 1823-06-01

**Authors:** 


*The Study of Medicine.* By John Mason Good, M.D. F.R.S.

[Continued from No. 12, page 832.]

The next subject which presents itself in the second volume, is the 4th order of the 3d class, Hæmatica, entitled Dysthetica, or Cachexies—a morbid state of the blood or blood-vessels, alone, or connected with a morbid state of the fluids. This order is divided into 9 genera, viz. Plethora, Hæmorrhagia, Marasmus, Struma, Carcinus, (cancer), Lues, Elephantiasis, Bucnemia, (tumid leg), Catacausis, (spontaneous combustion), Porphyra (scurvy), Exangia (vascular divarication), Gangrena, ulcus.

This is a pretty plentiful bill of fare, and may well furnish food for reflection.

Our author justly wonders that plethora is so seldom ranked as a disease, and so seldom treated of in a course of medical instruction. Assuredly it is a phenomenon that very frequently crosses our path in practice—perhaps more frequently than almost any other. Dr. Good properly enough divides plethora into two kinds—sanguineous and serous—the first more common to men, the second to women—the first characterized by a full, strong, rebounding pulse, firm and vigorous muscular fibres—the second by full, but frequent and feeble pulse, languid vital actions, smooth and soft skin, plump but inexpressive figure. In the sanguineous plethora, slight excitements produce congestions in the larger vessels or organs. The head feels heavy—the sleep is disturbed—the respiration is laborious. If fever arise it will assume the inflammatory type, and slight excesses endanger apoplexy.

The treatment of this species is well known—abstinence and depletion.

In the serous plethora there is little vigour or activity of any kind, and whatever is eliminated is suffered to accumulate;—hence costiveness is a common symptom—the ankles are cold and œdematous—fat accumulates in the cellular membrane—there is inertness of mind as well as body—every exertion is a fatigue, and the countenance though plump is without expression. The following are our author's therapeutics in this species of plethora. It is defective in eccoprotics and alterative aperients, which open the secretions and relieve the plethora. In other respects the advice is judicious.

"In this species, as in the last, we are compelled to begin with cupping or the use of the lancet. But, though the distended and overflowing vessels demand an abstraction of blood, it should never be forgotten that the relief hereby afforded is only temporary; and that, as the disease is, in this case, an effect of debility, we are directly adding to the cause as often as we have recourse to the lancet. Our leading object should be to give tone to the relaxed fibres; and to take off the morbid tendency to the production of a surplus of blood by counteracting the irritability which gives rise to it. Our attack must be made upon the entire habit, which as far as possible should undergo a total change. The diet should be nutritious, but perfectly simple, and the meals less frequent or less abundant than usual; the sedentary life should give way to exercise, at first easy and gentle, but by degrees more active, and of longer extent or duration. Tonics, as bitters, astringents and sea-bathing, may now be employed with advantage; and the muscular fibres will become firmer as the cellular substance loses its bulk.

"The whole, however, must be the work of time; for although in morals it is a wholesome principle that bad habits cannot too speedily be thrown off, it is a mischievous doctrine in medicine. Health being the middle term between excess and deficiency, every day is giving us a proof that where either of these extremes has become habitual, the system can only be let up or let down by slow degrees so as to reach and rest at the middle point with certainty, and without inconvenience. Professor Monro has furnished us with several very striking examples of this fact; and particularly among those who had acquired a habit of drinking very large quantities of spirituous potation. A man of this description who had broken both bones of one leg, and was put, for a more speedy recovery, upon a diet of milk and water and water-gruel, was hereby thrown into a low fever with an intermitting pulse, twitching tendons, and delirium; during which he got out of bed and kicked away the box in which his leg was confined. A by-stander and friend of the patient's of the same irregular habit, ventured to tell the Professor that he would certainly kill him if he did not allow him ale and brandy; since, for several years antecedently, he had been accustomed to both these as his common drink; a little of each was, in consequence, permitted him; but the patient's friends did not tie him down to this little; for extending the grant of an inch to an ell, they instantly gave the man a Scot's quart of ale and a gill of brandy, which was his usual allowance for the evening, he slept well and sound; the next morning was free from delirium and fever; and, by a perseverance in the same regimen, obtained a speedy cure without the least accident." 685.

Hæmorrhage is divided, as usual, into entonic and atonic, or active and passive; and some interesting remarks are made by our author on the vacillations of balance in the circulation at different periods of our existence.

In the article hæmoptysis, no notice is taken of the pathological state of the lungs, nor are the interesting researches of Læennec, or indeed of any of the recent pathologists at all adverted to. After premising venesection, the following is the methodus medendi recommended by Dr. Good.

"In general, however, we shall find it as successful and far less distressing to employ mild aperients and sedatives. The first, and particularly neutral salts, are alone of great benefit, and their action should be steadily maintained. Sedatives are of still higher importance, and especially those that reduce the tone of the circulation, as nitre and digitalis. The first, in about ten grains to a dose, should be given in iced water, and swallowed while dissolving; the dose being repeated every hour or two according to the urgency of the case. If there be much cough, it must be allayed by opium and blisters. Local astringents we cannot use, and general astringents are here manifestly counter-indicated, however useful in passive hemorrhage: though it should be recollected, that when an active hemorrhage from the lungs is profuse and obstinate, the vessels lose their tone and fall into a passive state." 697.

We observe that the superacetate of lead, one of the most powerful medicines we possess in restraining active as well as passive hæmorrhage from the lungs, is not alluded to by our author, except in the atonic species. We can positively assert that in all hæmorrhages from the lungs, the superacetate is a valuable medicine, in conjunction with opium, and after venesection (in the active species) has checked the vascular tumult. Our author has properly brought forward the testimonies of Dr. Reynolds and Dr. Latham to the good effects of the remedy in question.

While treating of marasmus, our author very judiciously introduces an abstract of Sir Henry Halford's interesting description of the "climacteric disease," published originally in the College Transactions, and now well known to the profession. Dr. Good has also introduced a case in illustration from his own practice, that of the late Mr. Cobb, secretary to the East India Company. But we are sorry to see the manner of relating this case. Of what interest is it to the pathologist or physician to be told, in a systematic work on medicine, of Mr. Cobb's "*extensive connexion and correspondence with the most brilliant and distinguished characters of the age that have figured either in political or fashionable life?*" What has the practitioner to do with Mr. Cobb's "fine taste and commanding talents?" Or, finally, what has science to do with a knowledge of the fact that—"His Royal Highness the Duke of Sussex was kind enough to accommodate our patient with the use of his easy and convenient sofa-carriage, for as long a period as he might choose?" We are astonished that a man of Dr. Good's taste and judgment would condescend to record such pompous inanities, or waste his own and his readers' time with things so much beneath the dignity of science.

On pulmonary consumption we did not expect that our author could throw any light—but we expected that Dr. Good would have brought down his information to the date of publication. This, however, he has not done—at least in an equable manner. Of the researches, for instance, of Laennec and Baron, he takes no notice, while the anti-phthisical "hurricane at Barbadoes," described by Sir Gilbert Blane, (though published long after Laennec and Baron,) and the "low vales of his own country," recommended in consumption by Mansford, are carefully placed before the reader's eye. This is passing strange!

On struma, cancer, and syphilis, we need not dwell. It is not a little remarkable that in a systematic view of the venereal disease, at the present day, no notice should be taken of the important investigations so long pursued by our army brethren in particular, respecting the possibility of curing the complaint without mercury. The following is the only passage that seems to have any reference to the subject. "Dr. J. Thomson, of the Depôt Hospital, Edinburgh Castle, has been so highly satisfied with its (sarsaparilla) antisyphilitic powers, that he has for some years relinquished the use of mercury altogether in favour of a new mode of practice which consists chiefly in the employment of sarsaparilla." In recompence for this unaccountable omission of one of the most interesting facts of the present century, Dr. Good enumerates the anti-venereal properties of the flammula jovis, the clematis recta, the lobilia syphilitica, and some other antiquated remedies!

On the subject of elephantiasis, our author is quite at home; as it has enabled him to thread the mazes of the Hebrew, Arabic, Egyptian, and Grecian languages, and, according to Dr. Bateman, "to disperse some of the thick mist in which the translators have enveloped them." As far as our own dull optics are concerned, this *mist* of antiquity seems still as thick as that which Ossian so often describes on the " Mountains of Morven." If these erudite researches respecting the *name* of the disease threw any light on its nature or treatment, we should be among the first to fall down and worship their author. But as " fine words butter no parsnips," neither will Hebrew characters disperse leprous tubercles—and therefore we are just where we started in the midst of learned lore.

In the article " scurvy," our author accumulates most of the knowledge that has been acquired respecting the disease by our forefathers, predecessors, and a few of our cotemporaries. He does not seem to be sufficiently acquainted with a fact established by our naval medical brethren, and particularly Mr. Bampfield, that the want of fresh *animal food* is as much the cause of scurvy as the want of vegetables—and also that fresh animal food will cure the disease as quickly and effectually as fresh fruits and acids. We understand that genuine scurvy has lately been pretty generally produced in the Penitentiary establishment at Milbank, by an experiment of feeding its inhabitants almost entirely on vegetables and the farinaceæ. It is probable, however, that in this instance, the low and damp situation of the building, the incarcerated condition of its inmates, and the gloomy depressing passions inseparable from such a life, must have materially contributed, with the banyan fare, to the production of scurvy, hemeralopia, &c. with which the inhabitants have been scourged.

In the remaining species of this order, we see nothing to find fault with, and the author has evinced his usual industry, erudition, and judgment in the compilation.

The third volume opens with the 4th class—Neurotica, embracing four orders, viz. 1st order, Phrenica affecting the intellect—Osthetica, the sensation—Cinetica, the muscles—Systatica, affecting several or all of the sensorial powers. The physiological proem to this class, as might have been expected, requires and occupies a considerable space—54 pages of letter-press, and does great credit to the ingenious author. The three principal heads of this proem are—1st, The general nature of the brain, its ramifications, and substitutes—2d. The principle of sensation and motion—3d. The intellectual principle.

In touching on the last and most intricate subject of the three, Dr. Good has wisely confessed the ignorance under which we labour, and properly recommended to us investigations more suited to our limited capacities.

" Of the nature of the mind or soul itself, we know little beyond what REVELATION has informed us: we have no chemical test that can reach its essence; no glasses that can trace its mode of union with the brain; no analogies that can illustrate the rapidity of its movements. And hence the darkness that, in this respect, hung over the speculations of the Indian gymnosophists and the philosophers of Greece continues without abatement, and has equally resisted the labours of modern metaphysicians and physiologists. That the mind is an intelligent principle we know from nature; and that it is a principle endowed with immortality, and capable of existing after death in a state separate from the body, to which, however, it is hereafter to be re-united at a period when that which is now mortal shall put on immortality, and death itself be swallowed up in victory —we learn from the God of nature. And with such information we may well rest satisfied: and, with suitable modesty, direct our investigations to those lower branches of this mysterious subject that lie within the grasp of our reason." 33,

Our author cannot, however, drop this subject without observing that the discussion concerning the particular entity of the mind, seems to have been conducted with an undue degree of heat and confidence on all sides, considering our ignorance of the subject.

"Is the essence of the mind, soul, or spirit, material or immaterial? The question, at first sight, appears to be of the utmost importance and gravity; and to involve nothing less that a belief or disbelief, not indeed in its divine origin, but in its divine similitude and immortality. Yet I may venture to affirm, that there is no question which has been productive of so little satisfaction, or has laid a foundation for wider and wilder errors within the whole range of metaphysics. And for this plain and obvious reason, that we have no distinct ideas of the terms, and no settled premises to build upon. Corruptibility and incorruptibility, intelligent and unintelligent, organized and inorganic, are terms that convey distinct meanings to the mind, and impart modes of being that are within the scope of our comprehension. But materiality and immateriality are equally beyond our reach. Of the essence of matter we know nothing, and altogether as little of many of its more active qualities: insomuch that, amidst all the discoveries of the day, it still remains a controvertible position, whether light, heat, magnetism, and electricity are material substances, material properties, or things superadded to matter, and of a higher nature.

"If they be matter, gravity and ponderability are not essential properties of matter, though commonly so regarded. And if they be things superadded to matter, they are necessarily immaterial, and we cannot open our eyes without beholding innumerable proofs of material and immaterial bodies co-existing and acting in harmonious union through the entire frame of nature. But if we know nothing of the essence, and but little of the qualities of matter, of that common substrate which is diffused around us in every direction, and constitutes the whole of the visible world, what can we know of what is immaterial? of the full meaning of a term that, in its strictest sense, comprehends all the rest of the immense fabric of actual and possible being; and includes, in its vast circumference, every essence and mode of essence of every other being, as well below as above the order of matter, and even that of the Deity himself?

"Shall we take the quality of extension as the line of separation between what is material and what is immaterial? This, indeed, is the general and favourite distinction brought forward in the present day; but it is a distinction founded on mere conjecture, and which will by no means stand the test of inquiry. Is space extended? every one admits it to be so. But is space material? is it body of any kind? Des Cartes, indeed, contended that it is body, and a material body; for he denied a vacuum, and asserted space to be a part of matter itself: but it is probable that there is not a single espouser of this opinion in the present day. If then extension belong equally to matter and to space, it cannot be contemplated as the peculiar and exclusive property of the former; and if we allow it to immaterial space, there is no reason why we should not allow it to immaterial spirit. If extension appertain not to the mind or thinking principle, the latter can have NO PLACE of existence; it can exist NO WHERE; for WHERE OR PLACE is an idea that cannot be separated from the idea of extension. And hence, the metaphysical immaterialists of modern time freely admit that the mind has NO PLACE of existence; that it does exist NO WHERE; while, at the same time, they are compelled to allow that the immaterial Creator, or universal Spirit, exists EVERY WHERE, substantially as well as virtually." 35.

Such are the difficulties in which our ingenious author has involved the immaterialists. But it is not to be supposed that these difficulties are to be removed by adding to matter the quality of solidity in conjunction with that of extension. We shall not, however, follow our author through this maze, but give his own creed in his own words—a creed, we think, as rational as any that can be offered for our acceptance in the present condition of our knowledge.

"Let not the author, however, be misunderstood upon this abstruse and difficult subject. That the mind has a DISTINCT NATURE and is a DISTINCT REALITY from the body; that it is gifted with immortality, endowed with reasoning faculties, and capacified for a state of separate existence after the death of the corporeal frame to which it is attached, are, in his opinion, propositions most clearly deducible from revelation, and, in one or two points, adumbrated by a few shadowy glimpses of nature. And that it may be a substance strictly IMMATERIAL and ESSENTIALLY DIFFERENT from matter, is both possible and probable; and will hereafter, perhaps, when faith is turned into vision, and conjecture into fact, be found to be the true and genuine doctrine upon the subject. But till this glorious era arrives; or till, antecedently to it, it be proved, which it does not hitherto seem to have been, that matter, itself of divine origin, gifted even at present, under certain modifications, with instinct and sensation, and destined to become immortal hereafter, is physically incapable, under some still more refined, exalted, and spiritualized modification, of exhibiting the attributes of the soul, of being, under such a constitution, endowed with immortality from the first, and capacified for existing separately from the external and grosser frame of the body; and that it is beyond the power of its own Creator to render it intelligent, or to give it even brutal perception, the argument must be loose and inconclusive: it may plunge us, as it has plunged thousands before, into errors, but can never conduct us to demonstration. It may lead us, on the one hand, to the proud Brahminical and Platonic belief, that the essence of the soul is the very essence of the Deity, and consequently a part of the Deity himself: or, on the other, to the gloomy regions of modern materialism, and to the cheerless doctrine that it dies and dissolves in one common grave with the body." 37.

But we must take leave of this subject, and of the proem altogether, to which we cannot do justice in a review.

The first order, (Phrenica, or diseases affecting the intellect,) is divided into six genera, viz. Insanity, Ungovernable Passion, Illusion, Revery, Sleep-disturbance, Fatuity. These are subdivided into species and varieties almost innumerable —in short, we should have imagined that the invention of so many names and distinctions would have required or produced phrenzy itself! Nevertheless, we freely confess that Dr. Good has accumulated on the subject of mental and nervous maladies an immense fund of curious and important information from the best sources, and which we can safely recommend to the careful perusal of the student in medical science.

Of the great variety of subjects treated of in the class Neurotica, our limits and plan will only permit us to take some slight notice of one or two.

The species Hypochondrias is neatly, though succinctly treated of by our author. The publication of M. Falret has brought the subject more before the public, of late, than many other diseases of greater importance. Assuredly, it is one which is equally distressing to patient and physician— for the former seldom acknowledges that he derives any benefit from the latter.

As early as the time of Diocles hypochondriacism was considered as a disease of the stomach, and notwithstanding the arguments of Falret, Georget, and some late writers, it does not seem to be yet satisfactorily settled whether it should be regarded as a cerebral or abdominal malady. For our own parts, we believe that in most cases there is functional disorder both of the brain and chylopoietic organs. It is not easy to distinguish hypochondriasis from melancholia, since, in fact, they often change into one another. Sir Alexander Crichton, however, lays down the following distinction :—" In those whose health is much deranged true melancholy seldom arises, except mental causes of grief and distress join themselves to the corporeal ones: and this constitutes one of the characters which distinguish melancholia *vera* from hypochondriasis. The *former* may be said to be always excited by mental causes, and consists in various phenomena of grief, despondency, and despair; whereas, the *latter* most commonly arises from corporeal causes, and its mental phenomena consist of erroneous ideas entertained about the patient's own make or body."*

" The corporeal causes" says Dr. Good, " are usually a diseased emotion of one or more of the digestive organs, and especially, as we shall presently have to observe, a displacement of some part of the colon. It is also not unfrequently a result of the sudden cessation of some periodical or other habitual discharge, as that of an issue, or of a hemorrhoidal flux, a chronic ulcer, or some external eruption.

" The melancholy man seldom lives long, and his disorder often commences in the meridian of life. He frequently terminates his days by violence, or at the utmost never attains old age. The hypo chondriac seldom becomes affected till after the meridian of life, and very generally continues to the stage of longevity." 146.

The common corporeal symptoms of hypochondriasis are a troublesome flatulence in the stomach or bowels—acrid eructations—costiveness—copious discharge of pale urine—spasmodic pains in various parts of the body—giddiness—dimness of sight—palpitations—sleeplessness—utter inability of fixing the attention upon any subject of importance, or of engaging in any thing that demands vigour or courage. The mental feelings and peculiar trains of ideas that haunt the imagination and overwhelm the judgment, exhibit an infinite diversity, and lay a foundation, Dr. Good thinks, for the three following diversities, viz. vapours—weariness of life—misanthropy, or spleen.

In the first variety the patient is tormented with a visionary or exaggerated sense of pains or some concealed disease—a whimsical dislike of particular times or places, or things—or groundless apprehensions of personal danger or poverty. Strange and manifold are the illusions under which men of otherwise excellent understandings have laboured in this respect. Greding gives us an account of a medical gentleman

---

* On Mental Derangement, vol. iii. p. 325.

who laboured under an impression that his stomach contained frogs, which had been successively spawning ever since he had bathed in a pool where some tadpoles had been swimming. He spent his time in trying to expel this imaginary evil, and had travelled to many places to consult eminent physicians. It was in vain to attempt to convince him that the gurglings or borborigmi he heard were from extricated wind in the stomach or bowels.

"I have, at this moment," says Dr. Good, "under my care, a hypochondriac of about fifty years of age, who affords sufficient proof that Moliere drew his Malade Imaginaire from nature, and hardly added an exaggerating touch. His profession is that of the law; his life has been uniformly regular, but far too sedentary and studious; without having any one clearly marked corporeal affection, he is constantly dreading every disease in the bills of mortality, and complaining, one after another, of every organ in his body; to each of which he points in succession as its seat: especially the head, the heart, and the testes. He now suspects he is going to have a cataract, and now frightens himself with an apprehension of an involuntary seminal emission. It is rarely that I have left him half an hour, but I have a note to inform me of some symptom he had forgotten to mention, and I have often five or six of these in the course of the day. The last was to state, that shortly after my visit he had had a discharge of three drops of blood from the nose—a change which he thought of great importance, and requiring immediate attention. His imaginary symptoms, however, soon disappear, provided they are listened to with gravity, and pretended to be prescribed for; but not otherwise. Yet in disappearing they only yield to others, that can only be surmounted in like manner. His head is too much confused to allow him to engage in any serious study, even if it were prudent to recommend it to him: but on all common subjects he is perfectly clear, and will converse with shrewdness and a considerable extent of knowledge. His bowels are sluggish; his appetite not good, though he eats sufficiently; his sleep is unquiet, but he has enough of it without opiates; his pulse is variable, sometimes hurrying on abruptly, and without any obvious cause, to a hundred strokes in a minute, but often very little quicker than in a state of health. His tongue varies equally, and is irregularly clean, milky, and brownish, and then suddenly clean again. He is irritable in his temper, though he labours to be calm; and is so rooted to his chamber, that it is difficult to drag him from it. He has now been ill about ten weeks, but it is during the winter, and the season is too severe and inclement for him to venture abroad. I look forward to his restoration in the spring from exercise, change of air, and a course of tonic medicines." 148.

It is a melancholy reflection that the wisest and best of mankind are as open to this affliction as the weakest—perhaps more so! Pascal himself was, at one time, so hallucinated with hypochondriacism as to believe that he was always on the verge of an abyss into which he was in danger of falling. Under the influence of this terror he would never sit down till a chair was placed on that side of him on which he thought he saw it, thus proving the floor to be substantial.

The second variety, weariness of life, ending often in suicide, we have sufficiently discussed in another part of this Journal.

Our author appears to think that in many cases of hypochondriacism there is a displacement of the colon, whether as cause or effect.

"Post-obit examinations have also frequently pointed out another local cause which otherwise we should little expect: and that is a displacement of the transverse colon. M. Pinel, as we have already observed, regards this as a very common cause of insanity in all its forms: but there can be no question that it is a powerful and ready cause of the present species of mental alienation. M. Esquirol, who has found it as frequently as M. Pinel, tells us that this displacement sometimes consists in an oblique, and sometimes in a perpendicular direction of the intestine, so that its sinister extremity lies behind the pubes; whilst it has sometimes descended into the form of an inverted aorta, even below the pubes and into the pelvis. No disease of the organization has been found in any instance, and hence the change of place must proceed from relaxation and debility alone, where the misposition is not connate; on which account it may, in some instances, be an effect, as it is certainly a cause in others. It is under these circumstances that we chiefly meet with that pain in the epigastrium to which we have already adverted, and which gives the feeling of a tight cord surrounding the body in the line of distress; and when such a symptom, therefore, occurs, we have reason to suspect the cause of the disease to be produced by some derangement of the colon, in respect to position. Under the operation of such a cause the art of medicine can do but little: temporary ease, however, may be obtained by the pressure of a belt broad enough to support the whole of the lower belly; and it is possible that the intestine may gradually right itself under a course of the warmer tonics, as columbo, canella alba and cassummuniar, or lose its morbid irritability by habit. But these are rare terminations; for more generally the displacement increases, and the disease itself gains ground and becomes more incurable." 153.

Congestions in one or more of the abdominal organs are a frequent result of this complaint—not seldom a primary cause. Hence we find that hæmorrhoidal discharges are often serviceable, and, on the same principle, that leeches to the anus afford much relief.

We have said that Georget, Falret, and some others (among whom indeed we may reckon Cullen himself) consider the brain as the seat of hypochondriacism, and conceive that the disorder of any other organ can seldom be regarded even as its remote cause. M. Falret,* who is the great modern champion of this doctrine, appeals to the phenomena and symptoms of the disease for proofs of its correctness. Hypochondriacs, says he, often complain of pain in the eyes, which organs appear occasionally to be larger during the paroxysms—they have some degree of intolerantia lucis—the arteries going to the head throb—the sense of hearing is acute, as are those of smelling, taste, and often of feeling—there is a predominant want of sleep—and when sleep occurs it is disturbed with frightful dreams. The intellect, at the beginning of the disease, is more acute than natural, but afterwards becomes confused. These, and many other symptoms which we have not thought it necessary to enumerate here, are looked upon by our author as indications that the sensorium is the seat of the disease.

The next order of symptoms M. Falret considers as sympathetic, though commonly looked upon by physicians as primary ones. These are, depravation of digestion, gastric acidity—often an inordinate flow of saliva from the mouth—sense of tightness in the region of the stomach and in the hypochondria—nausea, and sometimes vomiting of blackish matters—constipation or diarrhœa—borborygmi—great flow of pale urine, &c. &c. These and many other anomalous symptoms will often continue for years, without any great loss of flesh, and without the friends or physician attaching much importance to what are considered imaginary complaints. But at length the patient begins to lose flesh and colour—he has palpitations of the heart, and, occasionally, intermissions of the pulse, and other more evident symptoms of actual disease.

In respect to *post mortem* examinations, M. Falret has not been very successful in establishing his doctrine. Lesions have been found in all parts of the body, and in none exclusively. Indeed, it is evident that Falret endeavours to establish his position rather by physiological and pathological reasoning than by anatomical demonstration.

In regard to treatment, M. Falret advises, and perhaps properly, that not much medicine is to be given. He seems to depend chiefly on moral management, as that which goes most directly to relieve the suffering sensorium. He recommends leeches to be occasionally applied behind the ears,

---

* See the excellent analysis of Falret's work on Hypochondriasis, in our respected cotemporaries, the Medical and Physical Journal, Medical Repository, and the 17th number of the Quarterly Journal of Foreign Medicine.

and cold applications to the head; at the same time, prescribing, (what is rarely obtained) exercise, amusements, cheerful company, change of occupation and scene, &c. With respect to the sympathetic disorders, he says laconically, " *sublata causa, tollitur effectus*." But this is fallacious; for when hypochondriasis does actually originate in sensorial disorder, the abdominal or other sympathetic affections that take place subsequently require to be particularly attended to, as they keep up the primary complaint.

Returning to Dr. Good, we observe that he considers opium as a very doubtful medicine in hypochondriasis, though strongly recommended by several respectable writers, and gladly resorted to by the unhappy patients themselves. Dr. Cullen was decidedly averse to it, and we have found, that where the bowels are obstipated, opium is injurious. At the same time, as sleeplessness is a common feature of the disease, we have often exhibited an anodyne at bed-time, in conjunction with an aperient or diaphoretic, without any injurious consequences, and with great solace to the patient.

Passing over a great many genera and species of mental and nervous affections, we come to NEURALGIA, of which our author has exhibited an imperfect history, as far, at least, as respects our own times. For example, neither the belladonna, nor the carbonate of iron are introduced into the methodus medendi, while some pages are taken up with the operation of cutting the nerve—a remedy which is now hardly ever resorted to—at least with success. Under the second species of this disease, neuralgia pedis, we have the following case.

" In calling, as I believe for the first time, the attention of the medical profession to this species, by introducing it into the volume of Nosology before the presentation of M. Chaussier's book to the world, I had my eye directed to a very marked case which had then lately occurred to me in a clergyman of this metropolis, about forty-five years of age, but otherwise in firm health and cheerful spirits. He had for many years been a victim to it. The paroxysms were short, and of uncertain recurrence, but so acute as nearly to make him faint, and at length compelled him to relinquish the duties of the pulpit, for which from his zeal and eloquence he was admirably qualified, but where he had frequently been obliged to break off with great abruptness from the unexpected incursion of a fresh paroxysm. The pain usually extended up the calf of the leg towards the knee, and ramified towards the toes in an opposite direction, and was usually compared by himself to that of scalding verjuice poured over a naked wound. The tibial branches of the popliteal nerve, and particularly the plantar twigs, seem in this species to have been the part chiefly affected, though it is probable that some of the offsets from the peroneal branch associate in some instance in the morbid action.

"Every therapeutic process that the art of medicine in the hands of the most experienced physicians of this metropolis could devise, was in this case tried in a long and tedious succession in vain. Sometimes external and sometimes internal preparations, or a tight ligature, appeared to afford a temporary alleviation, and to protract the intervals: but never any thing more. It was in consequence proposed by a surgeon of great eminence to amputate the leg, which was at one time on the point of being submitted to, though protested against by the present author, on two accounts. First, the uncertainty whether the morbid condition of the nerve might not be seated chiefly in the origin instead of in the extremity of the nerve; in which case, amputation could be of no avail; and secondly, the chance that in process of time the keen sensibility of the affected branches would be worn out and obtunded by the violence of the action. Such was the undecided and miserable condition of this patient at the time of noticing his case on the publication of the author's volume of Nosology. Since this period, the prediction that the disease would gradully wear itself out, has been completed: the paroxysms are now slight and tolerable, and the intervals much longer; and the patient has for nearly a twelvemonth been able to resume the duties of his profession without any interruption." 296.

We think with our author that it would have been very wrong to have proceeded to amputation in the above case, as we have no doubt that the disease would have returned after the operation as bad as ever. Our improved pathology of the disease, now leads to look on neuralgia as a general rather than a local disorder, and consequently more success has been derived from constitutional than topical treatment. Our author has introduced a third species—neuralgia mammæ—but in fact, there is no part of the body which is not liable to the disease, and therefore the species might be multiplied ad infinitum. The following case, however, is interesting.

"It is about two years ago that I was requested by an eminent surgeon of this metropolis in dispensary practice, to examine a young woman, then eighteen years of age, who, for more than two years had been subject to a painful disorder of the breast, that seemed equally to defy all parallel and all mode of treatment. On examining into the nature of the symptoms, I found them as described in the preceding definition. The organ was full-formed, soft, and globular, without the slightest degree of inflammation, or hardness. When the paroxysm of pain was not present, it would bear pressure without inconvenience, but, during the pain, the whole breast was acutely sensible. The paroxysms returned at first five or six times in the course of the day, and were short and transient: but as the disease became more fixed, it became also more severe and extensive: for the agonizing fits at length recurred as often as once an hour, and sometimes more frequently: and from being comparatively concentrated, the lancinating shoots darted both downward in the course of the circumjacent ribs, and upwards to the axilla, whence they afterwards descended to the elbow, below which I do not know that they proceeded at any time. These fits were at length so frequent and vehement as to embitter her whole life, and incapacitate her from pursuing any employment, for it frequently happened that, if she attempted needle-work, her fingers abruptly dropped the needle a few minutes after taking hold of it, from a mixture of pungent pain and tremulous twitching. The twitching or snatches in the shoulder, for it at length reached to this height, were at one time so considerable as to give the patient an idea, to use her own words, that something was alive there; while, though the lancinating pain did not descend below the elbow, a considerable degree of trepidation reached occasionally to her fingers' ends. Her general health was in the mean time unaffected, and she was regular in menstruation.

"I had no hesitation in regarding this a non-descript species of neuralgia; and as little in communicating my fears that no plan of medicine we could lay down would be more than palliative, even if it should prove thus far beneficial, and that we must trust to time alone for a cure, and that obtuseness of sensibility which I have already noticed as a common consequence of high nervous irritation, continued till the organ becomes exhausted and torpefied.

"Every remedial process was, nevertheless, tried in succession for the purpose of obtaining relief, if not full success. Bleeding, local and general, frequently and profusely repeated; purgatives of all kinds; tonics and antispasmodics of all kinds; the hot and cold bath; electricity and galvanism in every form; rubefacients, blisters, setons, issues, and whatever else could be suggested, were enlisted into service in succession. But every thing was equally without avail: nor do I know that even a temporary relief was obtained by any of these. Narcotics of all kinds proved impotent: drowsiness, indeed, and a comotose stupor were hereby in various instances obtained, but the interval of wakefulness was as much as ever tormented with the same racking paroxysms. From the powerful influence of nux vomica in many cases of nervous affection, to some of which we shall have occasion to advert hereafter, I had some hope of producing a slight impression on the nerves affected: but the hope proved illusory: she took it in infusion as far as to about eight grains at a dose, three or four times a day, till her head was intolerably confused, and every other part become numb, but the paroxysms were intractable." 299.

The poor sufferer tried one dispensary after another, and from each was discharged as incurable. At length she began to improve without any other medicine than a seton under the breast.

This fourth class of nervous affections affords us no scope for reflection or matter for extraction. The subjects are pretty fairly handled, but present little above or below par for commentary. Among the numerous species of functional disorders of the nervous system, our attention was arrested for a moment on one which is rarely treated of in systematic works, or indeed considered as a disease at all. This is dysphoria simplex, or, in common language, "*fidgets.*" It is really a most disagreeable and troublesome complaint while it lasts, and often puzzled us to account for its origin and cause. The import of the term, as every one knows, is restlessness, unsteadiness, and perpetual change of place; and most languages have some peculiar word to express this annoying and irritable sensation.

"The actual cause" says Dr. Good, "seems to consist in an undue accumulation of sensorial power, which seeks an outlet, so to speak, at every pore, for want of a proper channel of expenditure. Thus every one becomes fidgety who is obliged to sit motionless beneath a long-drawn and tedious story of common-place facts totally destitute of interest: and still more so when he is eagerly waiting, and fully bottled up, as it were, to reply to an argument loaded with sophisms, absurdities, or untruths, and over which he feels to have a complete mastery. So the high-mettled horse is fidgety that, called out, in full caparison, and still restrained in his career, is panting for the race or the battle. 'So the squirrel, when confined in a cage, feels,' as Dr. Darwin has ingeniously observed on this disease which he calls jactitatio. 'a restless uneasiness from the accumulation of irritative power in his muscles, which were before in continual and violent exertion from his habit of life, and in this situation finds relief by perpetually jumping about his cage to expend a part of his redundant energy. For the same reason children that are constrained to sit in the same place at school for hours together, are liable to acquire a habit of playing with some of the muscles of their face, or hands, or feet, in irregular movements which are called tricks, to exhaust a part of the accumulated irritability by which they are goaded.'

"In the two last instances this irritability is simply accumulated for want of a proper outlet, and not from inordinate secretion. In the two preceding cases of the restrained horse and the restrained orator, there is added to this simple accumulation, for want of disbursement, an accumulation also from inordinate excitement." 467.

That the cause, in many cases, may be an undue accumulation of sensorial power, we are not inclined to deny; but we have found, in ourselves, that this fidgety state which usually assails us at night, is most certainly owing to something irritating the nerves of the primæ viæ, as cheese, pickles, or any kind of food that happens not to be digested properly in the stomach and duodenum. Green tea produces the same effect. To allay the nervous irritation thus induced, we have often been obliged to get out of bed, and walk the room for a quarter or half an hour, and that repeatedly in the same night.

The succeeding species, dysphoria anxietas—defined thus:—"the restlessness chiefly affecting the præcordia, with depression of spirits, and a perpetual desire of loco-motion," engaged our notice, as being a most disagreeable sensation, and better described or understood perhaps, by the simple terms gloom or horrors. We mention it because we think it is very often produced by bile (particularly of a depraved quality) in the primæ viæ. We have known this gloom or despondency removed in a very few minutes by a discharge of bile; and we generally take measures to clear the primæ viæ whenever we feel these "blue devils" approach us.

The more important subjects comprehended in the remaining part of this 3d volume, are apoplexy and epilepsy, which have been very fully treated of in this journal, when reviewing certain monographs on those particular topics. It is therefore unnecessary for us to enter on them here, especially as they are not discussed on a very large scale, and comprehend only the more common matters of fact as yet ascertained respecting them.

The 4th volume is occupied with the 5th class, GENETICA, diseases of the sexual function. The first order, Cenetoca, affecting the fluids; the 2d Orgastica, affecting the orgasm; the 3d, Carpotica, affecting the impregnation.

The physiological proem gives our learned and ingenious author an opportunity of illustrating his subject by a pretty general though succinct view of the mysterious process of procreation in the other tribes of animals besides man. But as the last volume embraces some interesting diseases of the genital organs, on which we mean to make some remarks, we shall construct a short article thereon for our next number.

